# Associated Factors and Latent Patterns of Work-Related Musculoskeletal Disorders Among Digestive Endoscopy Nurses: A Multicenter Cross-Sectional Study

**DOI:** 10.3390/healthcare14152369

**Published:** 2026-08-03

**Authors:** Qingqian Lu, Yi Liu, Yue Chen, Jinchuan Deng, Xiumei Li, Haixing Wang, Liang Wang

**Affiliations:** 1 Department of Endoscopy, The First Affiliated Hospital of Xiamen University, School of Medicine, Xiamen University, Xiamen 361003, China; lqq211306@outlook.com (Q.L.); dr_jeremy@163.com (Y.L.); dengjc123@outlook.com (J.D.); li.xiumei2026@outlook.com (X.L.); 2 Day Surgery Center Ward, The First Affiliated Hospital of Xiamen University, School of Medicine, Xiamen University, Xiamen 361003, China; xychenyue916@163.com

**Keywords:** digestive endoscopy nurses, associated factors, prevalence, latent class analysis, work-related musculoskeletal disorders, ergonomics, cross-sectional study

## Abstract

**Background:** Work-related musculoskeletal disorders (WMSDs) have been reported to be very common among hospital workers and particularly nurses. They are assumed or found to be a result of physical workload or poor posture at work and only secondarily a consequence of (general) stress. Due to the increasing workload, more and more digestive endoscopy nurses have WMSDs, but there is still a lack of comprehensive research on the specific factors and patterns of WMSDs in China. **Methods:** A cross-sectional study was conducted among 400 digestive endoscopy nurses from 35 cities across 10 provinces in China. Data were collected using the Nordic Musculoskeletal Questionnaire (NMQ) and analyzed using multivariable logistic regression and latent class analysis (LCA) to identify associated factors and WMSD occurrence patterns. **Results:** The overall 12-month prevalence of WMSDs was high (82.5%), particularly in the neck, shoulder, and lower back. Frequent participation in complex endoscopic procedures (adjusted OR range 1.6–1.7) and 6–10 years of work experience (adjusted OR range 1.7–2.5)—rather than daily procedure volume alone—were independently associated with an increased likelihood of WMSDs (*p* < 0.05). BMI, analyzed using three categories (<24, 24–27.9, ≥28 kg/m^2^), was not significantly associated with WMSDs in any body region. The LCA revealed three distinct WMSD patterns characterized by multisite pain: a Full-body Pain group (19.0%), a Neck–Shoulder–Lower Back–Upper Back Pain group (42.25%), and a Mild Pain group (38.75%). **Conclusions:** This study found that digestive endoscopy nurses were at high risk for WMSDs, mainly due to repetitive physical strain and ergonomic challenges. As this was a cross-sectional study, the reported associations should not be interpreted as causal. Ergonomic training, improved workstation design, and workload optimization are important measures to ensure nurses’ health and improve the quality of care.

## 1. Introduction

Work-related musculoskeletal disorders (WMSDs) are among the most prevalent occupational health problems worldwide, affecting nearly all sectors of the healthcare industry. They are characterized by pain or dysfunction of the muscles, tendons, and joints caused by repetitive movements, awkward postures, or excessive biomechanical loading [1,2]. The increasing burden of WMSDs not only impairs the physical health and job satisfaction of doctors and nurses but also reduces procedural efficiency and compromises patient safety, which has gained growing attention in occupational health management.

Over the past decade, the global expansion of gastrointestinal endoscopy, driven by the rising incidence of gastrointestinal malignancies and widespread adoption of minimally invasive procedures, has markedly increased the workload of endoscopy staff [3,4]. Studies have reported that 62–86% of gastrointestinal endoscopy personnel experience musculoskeletal pain during their careers, with the neck, shoulders, and lower back being the most affected regions [5,6].

Among these professionals, nurses have been reported in prior comparative studies to show a higher prevalence of WMSDs than physicians, plausibly reflecting prolonged static postures, repetitive upper-limb exertion, and high procedural intensity required during endoscopic examinations and therapeutic interventions [7,8,9].

However, most existing research has relied on single-site symptom analyses or focused narrowly on upper limb disorders, providing limited insight into the complex patterns and co-occurrence of multisite pain [10,11,12]. Furthermore, many studies have small sample sizes or are regionally constrained, limiting their generalizability to broader populations. Consequently, the comprehensive mechanisms underlying WMSDs in digestive endoscopy nurses, encompassing biomechanical, organizational, and psychosocial dimensions, remain insufficiently explored. Pooled estimates from recent systematic reviews and meta-analyses of musculoskeletal disorders among surgeons and general nursing staff report comparably high overall and body-region-specific prevalence, supporting the view that digestive endoscopy nursing shares core ergonomic risk exposures with these related occupational groups [13,14,15,16].

To address these gaps, this study introduces an innovative approach by applying latent class analysis (LCA) to identify distinct patterns of WMSD occurrence among nurses who perform digestive endoscopy. Specifically, prior single-site or small-sample studies cannot capture whether musculoskeletal symptoms co-occur in identifiable multisite patterns, limiting the design of targeted prevention strategies. Existing endoscopy-specific ergonomic guidance (e.g., the American Society for Gastrointestinal Endoscopy ergonomics guideline) emphasizes workstation adjustability and procedural micro-breaks, but has not been informed by symptom-cluster analyses such as LCA. Unlike traditional univariate analyses, LCA enables the identification of hidden subgroups characterized by specific symptom distributions, thereby revealing underlying syndromic structures and risk profiles [17]. By integrating LCA with multivariable logistic regression, this study advances the understanding of how occupational factors, such as procedural complexity, workload, body mass index (BMI), and professional experience, interact to shape musculoskeletal health outcomes. This multidimensional analytic framework reflects a shift toward precision-based ergonomics and individualized prevention strategies in occupational healthcare.

Therefore, this multicenter cross-sectional study aimed to investigate the prevalence, associated factors, and latent symptom patterns of WMSDs among digestive endoscopy nurses in China. The findings of this study are expected to provide a theoretical and empirical foundation for developing targeted ergonomic interventions, optimizing workload management, and enhancing the overall occupational well-being of endoscopy nursing staff. Unlike prior LCA applications to general occupational populations [17,18], this study is the first to apply LCA specifically to procedural exposures unique to digestive endoscopy nursing (participation in complex, multi-stage procedures such as ESD/ERCP), allowing symptom clusters to be linked to endoscopy-specific occupational determinants rather than general workplace factors. (Population: digestive endoscopy nurses; Exposure: occupational/procedural factors; Comparator: nurses with lower procedural exposure; Outcome: 12-month WMSD prevalence and latent symptom patterns.)

## 2. Materials and Methods

### 2.1. Study Design and Participants

This multicenter cross-sectional study was conducted using an anonymous online survey targeting digestive endoscopy nurses working in hospitals across China. Digestive endoscopy nurses from 35 cities in 10 provinces were invited to participate in the study. Participants were recruited via convenience and snowball sampling: the survey link was distributed by head nurses in endoscopy departments across the 35 cities in 10 provinces through professional nursing networks. A total of 401 questionnaires were completed; after excluding one response with incomplete or logically inconsistent data during quality control, 400 valid questionnaires were retained for analysis (valid-response rate: 99.75%). Because the survey was anonymous and no closed list of eligible nonrespondents existed, a comparison between responders and nonresponders was not possible, and a denominator-based invitation response rate could not be calculated.

The inclusion criteria were as follows: (1) age 18–60 years; (2) at least one year of experience in endoscopy nursing; and (3) possession of a valid nursing qualification certificate. The exclusion criteria were as follows: (1) a history of musculoskeletal trauma or surgery within the past year; or (2) congenital, obstetric, gynecological, or other nonoccupational musculoskeletal disorders.

### 2.2. Ethical Considerations

This study complied with the ethical principles of the Declaration of Helsinki. The research protocol was reviewed and approved by the Ethics Committee of the First Affiliated Hospital of Xiamen University ([2025] Scientific Research Ethics Review No. (137)). Before accessing the questionnaire, potential participants were provided with an online information sheet explaining the study’s purpose, procedures, voluntariness of participation, and data confidentiality. Electronic informed consent was obtained from all participants prior to survey completion by requiring them to click an “I agree” button. Participation was voluntary and anonymous. No personally identifiable information was collected, and each IP address was allowed to submit only one response to ensure data integrity and avoid duplicate responses.

### 2.3. Research Instruments

Data were collected using a structured self-administered questionnaire based on the standardized Nordic Musculoskeletal Questionnaire (NMQ) [19]. The Chinese version of the NMQ has demonstrated good test–retest reliability in nursing populations, with Kappa coefficients ranging from 0.72 to 1.00 [20]. The Chinese version of the Nordic Musculoskeletal Questionnaire (NMQ) was used without modification to assess musculoskeletal symptoms in nine anatomical regions. No changes were made to its wording, response options, anatomical regions, recall periods, or scoring procedures. Questions related to digestive endoscopy nursing tasks, including participation in complex endoscopic procedures, were collected separately in the general characteristics and occupational exposure section and were not incorporated into the NMQ.

### 2.4. Assessment Items

#### 2.4.1. WMSD Symptoms

Following the NMQ framework, WMSDs were assessed in nine anatomical regions: neck, shoulders, upper back, lower back, forearms/elbows, wrists/hands, hips/thighs, knees, and ankles/feet. For each region, participants were asked whether they had experienced musculoskeletal pain or discomfort that they believed was related to their work in the previous 12 months (yes/no). In this study, the presence of WMSD in a given body region was operationally defined as a positive response to the 12-month symptom item. These nine binary indicators were also used as input variables in the latent class analysis. WMSD status in this study therefore reflects self-reported, self-attributed musculoskeletal symptoms over the preceding 12 months and does not represent clinically confirmed diagnoses. Self-report over a 12-month recall window is subject to recall bias, and participants’ attribution of symptoms to work is subject to attribution bias, both of which may bias prevalence estimates in either direction.

#### 2.4.2. Demographic and Occupational Characteristics

The demographic variables included sex, age (years), height (cm), and weight (kg). Body mass index (BMI) was calculated as weight (kg) divided by height squared (m^2^) and analyzed categorically using three standard Chinese cut-points (<24, 24–27.9 [overweight], ≥28 [obese] kg/m^2^). Across all nine body regions, BMI category was not significantly associated with WMSDs in univariate analysis (all *p* > 0.05; Supplementary Tables S1–S9) and was therefore not retained in any multivariate model, including for the concurrent multisite pain outcome (Supplementary Table S10).

Occupational variables included years of work experience in endoscopy, daily endoscopy workload (number of patients assisted per day; categorized as <25 vs. ≥25 patients/day), hospital grade (secondary/primary vs. tertiary), education level (secondary school, junior college, bachelor’s degree, postgraduate), nursing administrative position (yes/no), nursing technical title (nurse, nurse practitioner, nurse in charge, associate chief nurse or above), and frequency of participation in complex endoscopic procedures (self-reported as “frequent” or “occasional”, without a fixed numeric threshold; acknowledged as a limitation, see Discussion). Complex procedures were defined as endoscopic submucosal dissection (ESD), endoscopic submucosal excavation (ESE), submucosal tunnelling endoscopic resection (STER), and endoscopic retrograde cholangiopancreatography (ERCP). Participation in such procedures was categorized as “frequent” or “occasional” based on self-reports.

### 2.5. Quality Control

The online survey system incorporated an automatic validation mechanism to prevent incomplete submissions. After data collection, logical consistency checks were performed by the research team in collaboration with the head nurses to identify and exclude responses with missing, inconsistent or invalid data.

### 2.6. Statistical Analysis

Statistical analyses were performed using the IBM SPSS Statistics (version 26.0, IBM Corp., Armonk, NY, USA). Continuous variables are expressed as medians (interquartile ranges), and categorical variables are expressed as frequencies and percentages. Statistical significance was set at *p* < 0.05. Prior to multivariable modelling, multicollinearity among candidate covariates (education level, nursing technical title, gender, hospital grade, age group, participation in complex endoscopic procedures, nursing administrative position, work experience, and BMI) was assessed using the generalized variance inflation factor (GVIF^1/(2×Df)); all values ranged from 1.042 to 1.340, well below the commonly accepted threshold of 2, indicating no problematic multicollinearity (Supplementary Figure S1). For each anatomical region and for the concurrent multisite pain outcome, multivariable logistic regression models included as the dependent variable the binary 12-month WMSD indicator; candidate covariates were selected via univariate screening (*p* < 0.05), and reference categories are specified in Supplementary Tables S1–S10. Latent class analysis (LCA) was conducted using Mplus (version 8.0, Muthén & Muthén, Los Angeles, CA, USA) to identify patterns of WMSD occurrence. Model selection was guided by the Akaike Information Criterion (AIC), Bayesian Information Criterion (BIC), adjusted BIC (aBIC), Lo–Mendell–Rubin (LMR) likelihood ratio test, and bootstrap likelihood ratio test (BLRT). Lower AIC, BIC, and aBIC values indicated better model fit, while LMR and BLRT *p*-values < 0.05 suggested that the k-class model was superior to the k–1 class model. Class membership was assigned using each participant’s maximum posterior probability (modal assignment). A conditional-probability threshold of 0.50 was used only to characterize each class’s dominant symptom profile for interpretive labelling, not for membership assignment. The three-class model was retained based jointly on the lowest BIC/adjusted BIC, the highest entropy (0.891) among the tested solutions, and the clinical interpretability of the resulting classes; this combined criterion is consistent with simulation evidence that BIC-based indices outperform likelihood-ratio-based tests (e.g., LMR) for class enumeration at moderate-to-large sample sizes [21]. As all exposure and outcome variables were self-reported within the same survey instrument, recall bias and common-method bias cannot be excluded and are acknowledged as limitations.

## 3. Results

### 3.1. General Characteristics of Participants

A total of 400 digestive endoscopy nurses were included in this study, of whom 352 (88.0%) were female and 48 (12.0%) were male. The median age of the participants was 35 years (IQR = 30.0–40.0). Most nurses (68.5%) worked in tertiary hospitals, while 31.5% were employed in secondary or primary healthcare institutions; the majority held a bachelor’s degree (69.0%), followed by junior college (27.5%), secondary school (2.5%), and postgraduate (1.0%) degrees. In terms of technical rank, 43.0% were nurses in charge, 37.3% nurse practitioners, 11.0% general nurses, and 8.7% Associate Chief Nurse or above. The median duration of work experience was 5.0 years (IQR, 2.5–9.0 years). More than half (60.5%) of the nurses frequently participated in complex endoscopic procedures, including ESD, ESE, STER, and ERCP (Table 1).

### 3.2. Prevalence of Work-Related Musculoskeletal Disorders (WMSDs)

**Figure 1:**
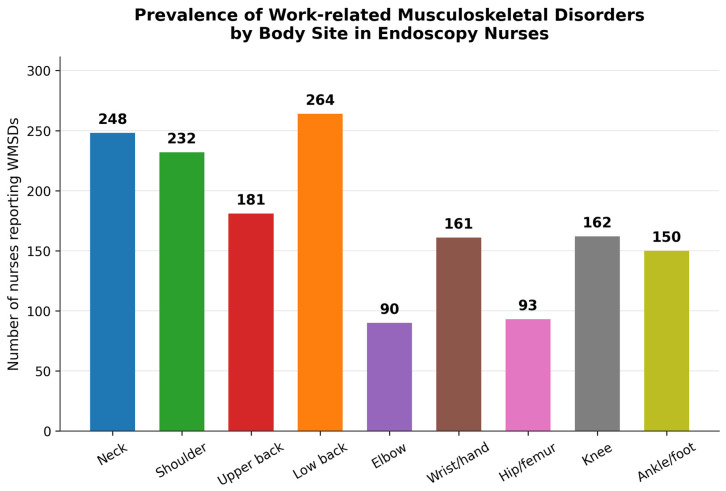
The WMSDS scores for all parts of the digestive endoscopy nurses.

The overall 12-month prevalence of WMSDs among the endoscopy nurses was 82.5%. The most commonly affected body regions were the lower back (66.0%), neck (62.0%), shoulder (58.0%), and upper back (45.25%). Moderate prevalence was observed in the knees (40.5%) and wrists/hands (40.25%), whereas the ankles/feet (37.5%), hips/thighs (23.25%) and elbows (22.5%) exhibited relatively lower prevalence rates. These findings revealed a widespread distribution of musculoskeletal symptoms among the endoscopy nurses (Figure 1).

### 3.3. Univariate and Multivariate Predictors of WMSDs

Logistic regression analysis identified several significant occupational factors associated with WMSDs. Models were fitted for all nine anatomical regions; Table 2 reports the four regions with at least one statistically significant independent predictor, while complete results for all nine regions, with explicit reference categories, are provided in Supplementary Tables S1–S9 (Table 2). Frequent participation in complex endoscopic procedures was significantly related to neck (OR = 1.61, 95% CI 1.05–2.47; *p* = 0.028) and shoulder (OR = 1.74, 95% CI 1.05–2.90; *p* = 0.033) pain. Nurses with 5–10 years of experience exhibited a higher risk of WMSDs in both the neck and shoulder regions (OR = 1.71, 95% CI 1.02–2.86; *p* = 0.041 and OR = 2.51, 95% CI 1.45–4.34; *p* < 0.001, respectively). Age was also significantly associated with the prevalence of shoulder (OR 2.29, 95% CI 1.19–4.40; *p* = 0.013) and knee pain (OR 4.75, 95% CI 1.74–12.97; *p* = 0.002), with nurses aged 41–50 years being more prone to experiencing pain. BMI, re-analyzed using three categories (<24, 24–27.9, ≥28 kg/m^2^) rather than the original two-category cutoff, was not significantly associated with shoulder pain (24–27.9 vs. <24: *p* = 0.057; ≥28 vs. <24: *p* = 0.171) or with any other body region (all *p* > 0.05; Supplementary Tables S1–S9), and was therefore not entered into any multivariate model. Collectively, these results highlight that age, work experience, and frequency of endoscopic procedures are key associated factors of WMSDs.

### 3.4. Latent Class Analysis (Lca) of WMSD Patterns

**Figure 2:**
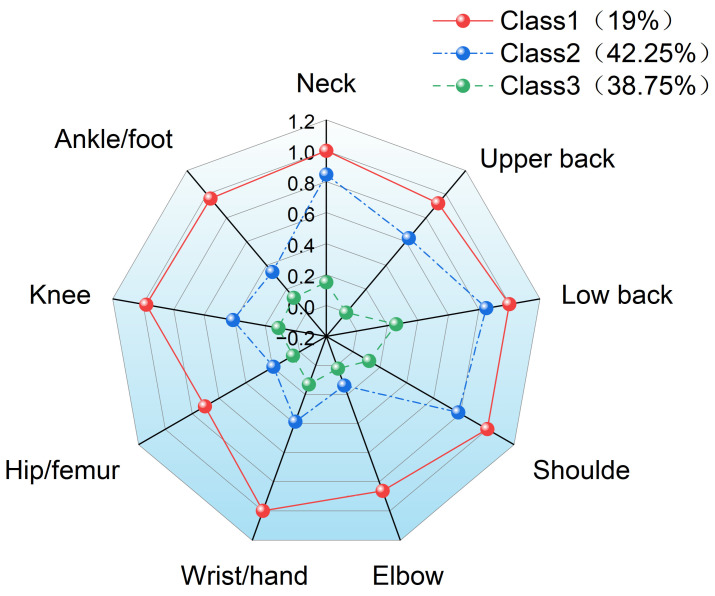
Conditional probability distribution of WMSDs patterns in digestive endoscopy nursing. Note: Class 1: Full-body pain Group; Class 2: Neck–Shoulder–Lower Back–Upper Back Pain Group; Class 3: Mild pain Group.

Latent Class Analysis (LCA) models with one to seven latent classes were tested sequentially to identify the optimal classification structure of WMSD symptom patterns among digestive endoscopy nurses. As the number of classes increased, the Akaike Information Criterion (AIC) decreased from the one-class to the three-class model but began to increase from the four-class model. The three-class model was therefore selected as the optimal solution, showing the lowest Bayesian Information Criterion (BIC) and the highest entropy value of 0.891. This indicates a high degree of classification precision and an adequate model fit, with the smallest class accounting for 19.0% of the sample (Table 3). Based on the three-class model, conditional probabilities for nine body regions (neck, shoulder, upper back, lower back, wrist/hand, elbow, hip/thigh, knee, and ankle/foot) were estimated and visualized using radar charts (Figure 2). A threshold of 0.50 for conditional probability was used to define the dominant symptom areas.

The three latent classes were interpreted and named as follows (Table 4): Class 1—Full-body Pain group (19.0%): High conditional probabilities in nearly all body regions, indicating widespread pain. Class 2—Neck–Shoulder–Lower Back–Upper Back Pain group (42.25%): High conditional probabilities (>0.5) in the neck, shoulders, lower back, and upper back, representing a multisite pain pattern. Class 3—Mild Pain group (38.75%): Low probabilities (<0.1) across all regions, representing occasional or mild discomfort. Given the observed 42.25% prevalence of the multisite pain pattern, this subgroup represents a substantial proportion of the workforce warranting targeted screening. Hospital grade and work experience are included as covariates in all regression models (Supplementary Tables S1–S10); formal interaction (effect-modification) testing was not conducted due to sample-size constraints and is proposed as a direction for future, larger studies.

### 3.5. Analysis of Influencing Factors for the Neck–Shoulder–Lower Back–Upper Back Pain Group

A total of 169 nurses (42.25%) were classified into the Neck–Shoulder–Lower Back–Upper Back Pain group, representing the most prevalent multisite WMSD pattern. Univariate analysis identified female sex, tertiary hospital level, participation in complex endoscopic procedures, work experience of 6–10 years, and BMI as potential associated factors. Multivariate logistic regression showed that participation in complex endoscopic procedures (OR = 1.62; 95% CI: 1.01–2.27; *p* = 0.025) and 6–10 years of work experience (OR = 2.12; 95% CI: 1.24–3.63; *p* = 0.006) were independent associated factors of membership in multisite (neck/shoulder/lower back/upper back) symptom groups (Table 5). Using the three-category BMI classification, neither the overweight (24–27.9 kg/m^2^, *p* = 0.360) nor the obese (≥28 kg/m^2^, *p* = 0.127) category was significantly associated with concurrent multisite pain relative to normal BMI (Supplementary Table S10).

## 4. Discussion

This multicenter cross-sectional investigation revealed a markedly high prevalence of work-related musculoskeletal disorders (WMSDs) among digestive endoscopy nurses in China, highlighting a significant occupational health challenge in this profession. We found that the most frequently affected anatomical regions were the neck, shoulders, lower back, and knees, aligning with prior reports among nursing and other healthcare professionals, which underscores the physical burden of endoscopy-assisted nursing [22,23,24]. The demanding nature of endoscopic nursing is characterized by frequent procedural participation, sustained static postures, repetitive upper limb exertion, and awkward body mechanics, rendering this population particularly vulnerable to musculoskeletal strain [5,6,25]. These findings emphasize that the cumulative physical load associated with high procedural intensity and prolonged standing may precipitate chronic tissue fatigue and inflammation over time.

The observed 12-month WMSD prevalence in this study was 82.5%, corresponding with global data that report rates of 62–86% among endoscopy personnel and up to 90% in nursing populations [26,27]. The somewhat higher prevalence in our cohort may reflect increased procedural volumes and elevated nurse-to-procedure ratios in Chinese tertiary and secondary hospitals, where endoscopic workload and patient turnover are substantially greater than in some European settings [28,29,30]. These differences may also be due to variations in workload quantification, ergonomic practice standards, staffing models, or cultural norms related to symptom reporting, indicating the need for context-specific ergonomic assessment.

Using latent class analysis (LCA), we identified three distinct symptom clusters: full-body pain, neck–shoulder–lower back–upper back pain, and mild-pain. The predominance of the first two classes is consistent with existing LCA-based categorizations of multisite pain among occupational populations [17,18]. The presence of widespread overlapping pain regions may reflect shared biomechanical pathways and potential central sensitization mechanisms, wherein prolonged static postures and repetitive motions induce local muscle ischemia and contribute to diffuse pain perception beyond the initial injury locus [31]. In our sample, approximately one-third of the participants experienced multisite pain, a figure aligned with the literature linking multisite WMSDs to greater disability, impaired work performance, and reduced quality of life [32]. Accordingly, this underscores the importance of conceiving WMSDs not as isolated regional phenomena but as interrelated systemic conditions that warrant integrated ergonomic and occupational health strategies.

Among the occupational determinants examined, frequent participation in complex multi-stage endoscopic procedures, including endoscopic submucosal dissection (ESD), endoscopic submucosal tumor excision (ESE), submucosal tunnelling endoscopic resection (STER) and endoscopic retrograde cholangiopancreatography (ERCP), was significantly associated with an increased WMSD risk. These procedures typically require nurses to assist with patient positioning, manipulate endoscopic equipment, apply manual abdominal compression, and sustain prolonged standing or leaning. Previous studies have similarly identified extended procedural durations, such as ERCP or colonoscopy, as factors associated with neck, shoulder, and back injuries [10,33]. Ergonomic deficiencies, such as fixed-height workstations, misaligned monitors, and non-adjustable equipment, further aggravate the loading on the cervical and lumbar spine [25,34]. Trunk flexion, torso rotation, and overhead reaching movements impose significant mechanical stress on the spine and shoulder girdle, contributing to cumulative microtrauma [35]. In addition, the combined effect of physical fatigue and psychological strain arising from long shifts and intense procedural demands may amplify the risk of WMSDs by increasing sustained muscle tension and impeding recovery [36].

Meanwhile, BMI was re-analyzed using three categories (<24, 24–27.9, ≥28 kg/m^2^) rather than the original two-category cutoff, following a reviewer’s suggestion. Under this finer categorization, BMI showed no statistically significant association with shoulder pain or with any of the other eight body regions (all *p* > 0.05; Supplementary Tables S1–S9). This indicates that an earlier, coarser two-category analysis had suggested an association with shoulder pain that did not replicate at finer categorization, illustrating the value of avoiding broad binary BMI cutoffs in occupational musculoskeletal research and cautioning against over-interpreting single-cutoff BMI findings in cross-sectional designs [37].

Female nurses also reported a higher WMSD prevalence than their male counterparts. This may reflect physiological differences in muscle strength, joint flexibility, and connective tissue characteristics, but could equally reflect differences in task allocation or ergonomic exposure between male and female staff, which were not measured in this study [1,38].

Work experience between five and ten years emerged as a significant predictor of WMSDs; mid-career nurses exhibited the highest prevalence of neck and shoulder pain, which may reflect cumulative exposure to repetitive strain before ergonomic adaptation or role transition occurs. Senior nurses may shift toward supervisory or administrative roles, thereby reducing the direct procedural load and illustrating a potential “healthy worker” effect.

The delineation of discrete pain clusters and associated factors provides actionable directions for tailored preventive strategies. Ergonomic interventions should prioritize workstation adjustability, including height-variable endoscopy towers, properly aligned monitors, and anti-fatigue floors. Limiting consecutive participation in complex procedures, incorporating micro-breaks, and implementing rotational staffing schedules may substantially reduce physical strain [34,39].

Workplace-based weight-management and psychological-support programs may be worth exploring in future intervention studies, though our cross-sectional data cannot establish their effectiveness, and the present analysis did not identify a robust independent BMI association. Female nurses who may be at an elevated risk for repetitive strain injuries could benefit from targeted exercise regimes designed to strengthen core and shoulder musculature. For mid-career and higher-experience nurses, periodic ergonomic reassessment and health monitoring should be emphasized in the workplace. Moreover, psychological support, such as stress-management workshops and counselling, may mitigate central sensitization in multisite pain clusters and bolster overall resilience [40,41].

Beyond conventional ergonomic redesign, structured self-management protocols incorporating slow controlled movements, breathing coordination, and postural correction exercises (e.g., yoga-based asana protocols) have shown promise for reducing neck, shoulder, and upper-back musculoskeletal symptoms in other occupational groups, and may warrant evaluation as an adjunct strategy in endoscopy nursing, pending intervention studies.

Institutional policies that support routine ergonomic audits and interdisciplinary collaboration involving occupational therapists, ergonomists, and endoscopy teams may standardize preventive practices and ensure the long-term sustainability of interventions.

This study is the first to apply LCA to identify distinct WMSD patterns among digestive endoscopy nurses in China, thereby offering a more refined understanding of symptom clustering beyond traditional region-specific analyses of WMSDs. The multicenter design and utilization of the Nordic Musculoskeletal Questionnaire enhanced generalizability and comparability of the results. Participants were mainly recruited from secondary and tertiary hospitals, which could reflect the diverse procedural volumes and staffing models typical of large medical institutions in China. However, the findings may not be fully generalizable to primary care or outpatient endoscopy units with lower caseloads or different ergonomic contexts.

Despite the aforementioned findings, there are still some limitations. First, the cross-sectional design precludes causal inference, and reliance on self-reported data may introduce recall or response bias. Because occupational exposures and WMSD symptoms were measured concurrently, this cross-sectional design cannot establish temporal precedence; it is possible that nurses experiencing musculoskeletal symptoms alter their procedural participation, rather than procedural participation causing symptoms. As all exposure and outcome variables were self-reported within the same survey instrument, recall bias and common-method bias cannot be excluded. Recruitment relied on convenience and snowball sampling rather than a closed invitee list, so self-selection bias and limited generalizability cannot be excluded. The standard LCA model assumes local independence of the nine symptom indicators conditional on class membership; given known biomechanical overlap between anatomically adjacent regions (e.g., neck–shoulder, lower back–upper back), residual local dependence cannot be excluded, as bivariate residual diagnostics were not obtained in the present analysis. Psychosocial work stress, individual physical fitness, and endoscopy-room ergonomic design were not measured and could not be adjusted for as covariates; residual confounding by these unmeasured factors is possible. Finally, the “frequent/occasional” procedural-participation classification relied on self-report without a fixed quantitative threshold. Additionally, unmeasured variables such as psychosocial factors, ergonomic training histories, or non-occupational physical activity may influence the observed associations. Further research should adopt longitudinal cohort designs to monitor the evolution of symptom clusters over time, integrate objective biomechanical assessments, such as motion capture and wearable sensors, and examine the effectiveness of ergonomic interventions in reducing cluster membership and WMSD incidence.

## 5. Conclusions

In conclusion, this study documented the high prevalence of WMSDs and heterogeneous symptom patterns among digestive endoscopy nurses. Procedural complexity and mid-career work experience were factors associated with multisite musculoskeletal strain. The LCA-derived clusters offer a novel descriptive framework that may inform, but does not yet validate, precision ergonomic strategies; prospective studies incorporating objective ergonomic and biomechanical measurements are needed to confirm these patterns and evaluate targeted interventions.

## Figures and Tables

**Table 1:** Demographic and Occupational Characteristics of the Digestive Endoscopy Nurses (*n* = 400).

Variable	Category	*n* (%) or Median (IQR)
Gender	Male/Female	48 (12.0)/352 (88.0)
Age (years)	–	35.0 (30.0–40.0)
BMI (kg/m^2^)	–	21.6 (19.7–23.5)
Work experience (years)	–	5.0 (2.5–9.0)
Hospital grade	Secondary/primary/Tertiary	126 (31.5)/274 (68.5)
Education level	Secondary/Junior college/Bachelor/Postgraduate	10 (2.5)/110 (27.5)/276 (69.0)/4 (1.0)
Nursing administrative position	No/Charge nurse	347 (86.7)/53 (13.3)
Nursing technical title	Nurse/Nurse practitioner/Nurse in charge/Associate Chief Nurse or above	44 (11.0)/149 (37.3)/172 (43.0)/35 (8.7)
Participation in complex endoscopic procedures	Frequent/Occasional	242 (60.5)/158 (39.5)

**Table 2:** Univariate and Multivariate Predictor Analyses of WMSDs among Digestive Endoscopy Nurses.

Body Part	Variable	Univariate OR (95% CI), *p*	Multivariate OR (95% CI), *p*
Neck	Participation in complex endoscopic procedures	1.70 (1.12–2.56), 0.012	1.61 (1.05–2.47), 0.028
Neck	Working experience (6–10 years)	1.87 (1.15–3.04), 0.012	1.71 (1.02–2.86), 0.041
Shoulder	Participation in complex endoscopic procedures	1.87 (1.25–2.82), 0.003	1.74 (1.05–2.90), 0.033
Shoulder	Working experience (6–10 years)	2.51 (1.54–4.09), <0.001	2.51 (1.45–4.34), <0.001
Elbow	Female gender	2.74 (1.05–7.13), 0.039	3.11 (1.01–9.58), 0.049
Knee	Participation in complex endoscopic procedures	1.70 (1.12–2.58), 0.013	1.69 (1.01–2.80), 0.044
Knee	Working experience (6–10 years)	2.08 (1.30–3.31), 0.002	1.85 (1.09–3.12), 0.022

Note: Complete results for all nine anatomical regions, including the BMI variable (re-analyzed with three categories and not significant in any region) are provided in Supplementary Tables S1–S9. The BMI–shoulder association reported in an earlier two-category analysis was not replicated at finer categorization and is therefore omitted from this table.

**Table 3:** Fit Statistics for Latent Class Models of WMSDs Patterns Among Endoscopy Nurses.

Classes	Parameters	AIC	BIC	aBIC	LMRT	BLRT	Smallest Class %	Entropy
1	9	4626.02	4661.94	4633.38				
2	19	3809.34	3885.17	3824.88	0.026	<0.001	46.5	0.827
3	29	3595.78	3711.53	3619.51	<0.001	<0.001	19.0	0.891
4	39	3563.50	3719.16	3595.41	0.052	<0.001	15.5	0.817
5	49	3558.74	3754.37	3598.84	0.104	0.1017	5.25	0.848
6	59	3557.24	3792.74	3605.52	0.044	0.2857	5.0	0.850

**Table 4:** Conditional Probabilities and Characteristics of Latent Classes.

Body Part	Class 1—Full-Body Pain Group (19%)	Class 2—Neck–Shoulder–Lower Back–Upper Back Pain Group (42.25%)	Class 3—Mild Pain Group (38.75%)
Neck	1	0.847	0.15
Upper back	0.924	0.629	0
Lower back	1	0.849	0.257
Shoulder	1	0.784	0.119
Elbow	0.863	0.138	0.021
Wrist/hand	1	0.386	0.13
Hip/femur	0.705	0.194	0.049
Knee	0.98	0.412	0.114
Ankle/foot	0.963	0.343	0.126

**Table 5:** Multivariate Logistic Regression for Concurrent WMSDs in the Neck, Shoulders, Lower Back and Upper Back.

Variable	Univariate OR (95% CI), *p*	Multivariate OR (95% CI), *p*
Female gender	1.22 (0.63–2.37), 0.548	–
Tertiary hospital	1.65 (1.03–2.63), 0.036	–
Participation in complex endoscopic procedures	1.72 (1.11–2.67), 0.016	1.62 (1.01–2.27), 0.025
Work experience 6–10 years	2.15 (1.33–3.48), 0.002	2.12 (1.24–3.63), 0.006
BMI 24–27.9 kg/m^2^ (vs. <24 kg/m^2^)	n.s., *p* = 0.360	–
BMI ≥ 28 kg/m^2^ (vs. <24 kg/m^2^)	n.s., *p* = 0.127	–

## Data Availability

The data presented in this study are available on request from the corresponding author. The data is not publicly available due to privacy or ethical restrictions.

## Supplementary Materials

The following supporting information can be downloaded at: https://www.mdpi.com/article/10.3390/healthcare14152369/s1, Figure S1: GVIF collinearity. Table S1. Univariate and multivariate logistic regression analysis of factors associated with Neck WMSDs. Table S2. Univariate and multivariate logistic regression analysis of factors associated with Upper back WMSDs. Table S3. Univariate and multivariate logistic regression analysis of factors associated with Low back WMSDs. Table S4. Univariate and multivariate logistic regression analysis of factors associated with Shoulder WMSDs. Table S5. Univariate and multivariate logistic regression analysis of factors associated with Elbow WMSDs. Table S6. Univariate and multivariate logistic regression analysis of factors associated with Wrist WMSDs. Table S7. Univariate and multivariate logistic regression analysis of factors associated with Hip WMSDs. Table S8. Univariate and multivariate logistic regression analysis of factors associated with Knee WMSDs. Table S9. Univariate and multivariate logistic regression analysis of factors associated with Ankle WMSDs. Table S10. Univariate and multivariate logistic regression analysis of factors associated with concurrent work-related musculoskeletal disorders (WMSDs) in the neck, shoulders, lower back, and upper back among digestive endoscopy nurses.
