# Supplementary material for: Associated Factors and Latent Patterns of Work-Related Musculoskeletal Disorders Among Digestive Endoscopy Nurses: A Multicenter Cross-Sectional Study
Source: Healthcare (Basel). 2026 Aug 3;14(15):2369. doi: 10.3390/healthcare14152369 (PMC13465618; doi:10.3390/healthcare14152369)
Supplement: Supplementary file 1 [file healthcare-14-02369-s001.zip › Supplementary Table S10 Concurrent Pain.pdf]

Table S10. Univariate and multivariate logistic regression analysis of factors associated with concurrent work-related musculoskeletal disorders (WMSDs) in the neck, shoulders, lower back, and upper back among digestive endoscopy nurses.

Variable	Category	n	Cases, n (%)	Univariate OR (95% CI)	P	Multivariate aOR (95% CI)	P
Hospital Grade	Secondary and primary hospitals (Ref.)	126	33 (26.2%)	1.00 (Reference)			
	Tertiary hospitals	274	99 (36.1%)	1.65 (1.03–2.63)	0.036		
Workload (patients/day)	<25 (Ref.)	127	54 (42.5%)	1.00 (Reference)		1.00 (Reference)	
	≥25	273	78 (28.6%)	0.54 (0.35–0.84)	0.006	0.56 (0.36–1.09)	0.073
Participation in complex endoscopic procedures	Occasional (Ref.)	158	41 (25.9%)	1.00 (Reference)		1.00 (Reference)	
	Frequent	242	91 (37.6%)	1.72 (1.11–2.67)	0.016	1.62 (1.01–2.27)	0.025
Age (years)	21–30 (Ref.)	115	33 (28.7%)	1.00 (Reference)			
	31–40	201	70 (34.8%)	1.33 (0.81–2.18)	0.264		
	41–50	68	24 (35.3%)	1.36 (0.71–2.57)	0.352		
	51–60	16	5 (31.2%)	1.13 (0.36–3.50)	0.833		
Gender	Male (Ref.)	48	14 (29.2%)	1.00 (Reference)			
	Female	352	118 (33.5%)	1.22 (0.63–2.37)	0.548		
BMI (kg/m²)	<24 (Ref.)	310	108 (34.8%)	1.00 (Reference)			
	24–27.9	72	21 (29.2%)	0.77 (0.44–1.35)	0.360		
	≥28	18	3 (16.7%)	0.37 (0.11–1.32)	0.127		
Nursing administration position	No (Ref.)	347	115 (33.1%)	1.00 (Reference)			
	Charge nurse	53	17 (32.1%)	0.95 (0.51–1.77)	0.878		
Nursing technical title	Nurse (Ref.)	44	14 (31.8%)	1.00 (Reference)			
	Nurse-in-charge	172	60 (34.9%)	1.15 (0.57–2.33)	0.702		
	Nurse practitioner	149	47 (31.5%)	0.99 (0.48–2.03)	0.973		
	Associate Chief Nurse or above	35	11 (31.4%)	0.98 (0.38–2.55)	0.970		
Work experience (years)	<5 (Ref.)	222	58 (26.1%)	1.00 (Reference)		1.00 (Reference)	

Variable	Category	n	Cases, n (%)	Univariate OR (95% CI)	P	Multivariate aOR (95% CI)	P
	6–10	111	48 (43.2%)	2.15 (1.33–3.48)	0.002	2.12 (1.24–3.63)	0.006
	>10	67	26 (38.8%)	1.79 (1.01–3.19)	0.047	1.66 (0.93–2.98)	0.089

Note: OR, odds ratio; aOR, adjusted odds ratio; CI, confidence interval. "Concurrent WMSDs" is defined as the simultaneous presence of work-related musculoskeletal symptoms in all four sites (neck, shoulders, lower back, upper back) (n = 132/400, 33.0%). Only variables reaching P < 0.05 in the univariate analysis (Workload, Participation in endoscopic procedures, Work experience) were entered into the multivariate model.
