# Supplementary material for: Associated Factors and Latent Patterns of Work-Related Musculoskeletal Disorders Among Digestive Endoscopy Nurses: A Multicenter Cross-Sectional Study
Source: Healthcare (Basel). 2026 Aug 3;14(15):2369. doi: 10.3390/healthcare14152369 (PMC13465618; doi:10.3390/healthcare14152369)
Supplement: Supplementary file 1 [file healthcare-14-02369-s001.zip › Supplementary Tables S1-S9.pdf]

Table S1. Univariate and multivariate logistic regression analysis of factors associated with Neck WMSDs.

Variable	Category	n	Cases, n (%)	Univariate OR (95% CI)	P	Multivariate aOR (95% CI)	P
Hospital Grade	Secondary and primary hospitals (Ref.)	126	71 (56.3%)	1.00 (Reference)			
	Tertiary hospitals	274	177 (64.6%)	1.41 (0.92–2.17)	0.115		
Workload (patients/day)	<25 (Ref.)	127	86 (67.7%)	1.00 (Reference)			
	≥25	273	162 (59.3%)	0.70 (0.45–1.08)	0.109		
Participation in endoscopic procedures	Occasional (Ref.)	158	86 (54.4%)	1.00 (Reference)		1.00 (Reference)	
	Frequent	242	162 (66.9%)	1.70 (1.12–2.56)	0.012	1.61 (1.05–2.47)	0.028
Age (years)	21–30 (Ref.)	115	66 (57.4%)	1.00 (Reference)		1.00 (Reference)	
	31–40	201	124 (61.7%)	1.20 (0.75–1.91)	0.453	1.08 (0.66–1.77)	0.748
	41–50	68	49 (72.1%)	1.91 (1.01–3.65)	0.049	–	–
	51–60	16	9 (56.2%)	0.95 (0.33–2.74)	0.931	0.81 (0.24–2.74)	0.735
Gender	Male (Ref.)	48	25 (52.1%)	1.00 (Reference)			
	Female	352	223 (63.4%)	1.59 (0.87–2.92)	0.134		
BMI (kg/m²)	<24 (Ref.)	310	197 (63.5%)	1.00 (Reference)			
	24–27.9	72	39 (54.2%)	0.68 (0.40–1.14)	0.141		
	≥28	18	12 (66.7%)	1.15 (0.42–3.14)	0.789		
Nursing administration position	No (Ref.)	347	215 (62.0%)	1.00 (Reference)			
	Charge nurse	53	33 (62.3%)	1.01 (0.56–1.84)	0.966		
Nursing technical title	Nurse (Ref.)	44	25 (56.8%)	1.00 (Reference)			
	Nurse-in-charge	172	112 (65.1%)	1.42 (0.72–2.78)	0.309		
	Nurse practitioner	149	89 (59.7%)	1.13 (0.57–2.23)	0.730		
	Associate Chief Nurse or above	35	22 (62.9%)	1.29 (0.52–3.19)	0.587		
Work experience (years)	<5 (Ref.)	222	124 (55.9%)	1.00 (Reference)		1.00 (Reference)	
	6–10	111	78 (70.3%)	1.87 (1.15–3.04)	0.012	1.71 (1.02–2.86)	0.041
	>10	67	46 (68.7%)	1.73 (0.97–3.09)	0.064	1.48 (0.73–3.00)	0.272

Note: OR, odds ratio; aOR, adjusted odds ratio; CI, confidence interval; WMSDs, work-related musculoskeletal disorders. Only variables reaching P < 0.05 in the univariate analysis were entered into the multivariate model; "–" indicates the variable/category was entered into the multivariate model but did not reach statistical significance; blank multivariate cells indicate the variable was not entered into the multivariate model.

Table S2. Univariate and multivariate logistic regression analysis of factors associated with Upper back WMSDs.

Variable	Category	n	Cases, n (%)	Univariate OR (95% CI)	P	Multivariate aOR (95% CI)	P
Hospital Grade	Secondary and primary hospitals (Ref.)	126	54 (42.9%)	1.00 (Reference)			
	Tertiary hospitals	274	127 (46.4%)	1.15 (0.75–1.76)	0.515		
Workload (patients/day)	<25 (Ref.)	127	64 (50.4%)	1.00 (Reference)			
	≥25	273	117 (42.9%)	0.74 (0.48–1.13)	0.159		
Participation in endoscopic procedures	Occasional (Ref.)	158	63 (39.9%)	1.00 (Reference)			
	Frequent	242	118 (48.8%)	1.43 (0.96–2.15)	0.081		
Age (years)	21–30 (Ref.)	115	45 (39.1%)	1.00 (Reference)			
	31–40	201	97 (48.3%)	1.45 (0.91–2.31)	0.117		
	41–50	68	33 (48.5%)	1.47 (0.80–2.69)	0.215		
	51–60	16	6 (37.5%)	0.93 (0.32–2.75)	0.900		
Gender	Male (Ref.)	48	18 (37.5%)	1.00 (Reference)			
	Female	352	163 (46.3%)	1.44 (0.77–2.67)	0.252		
BMI (kg/m²)	<24 (Ref.)	310	146 (47.1%)	1.00 (Reference)			
	24–27.9	72	28 (38.9%)	0.71 (0.42–1.21)	0.209		
	≥28	18	7 (38.9%)	0.71 (0.27–1.89)	0.499		
Nursing administration position	No (Ref.)	347	157 (45.2%)	1.00 (Reference)			
	Charge nurse	53	24 (45.3%)	1.00 (0.56–1.79)	0.996		
Nursing technical title	Nurse (Ref.)	44	17 (38.6%)	1.00 (Reference)			
	Nurse-in-charge	172	83 (48.3%)	1.48 (0.75–2.91)	0.255		
	Nurse practitioner	149	65 (43.6%)	1.23 (0.62–2.44)	0.557		
	Associate Chief Nurse or above	35	16 (45.7%)	1.34 (0.54–3.29)	0.527		
Work experience (years)	<5 (Ref.)	222	92 (41.4%)	1.00 (Reference)			

Variable	Category	n	Cases, n (%)	Univariate OR (95% CI)	P	Multivariate aOR (95% CI)	P
	6–10	111	55 (49.5%)	1.39 (0.88–2.19)	0.161		
	>10	67	34 (50.7%)	1.46 (0.84–2.52)	0.179		

Note: OR, odds ratio; aOR, adjusted odds ratio; CI, confidence interval; WMSDs, work-related musculoskeletal disorders. Only variables reaching P < 0.05 in the univariate analysis were entered into the multivariate model; "–" indicates the variable/category was entered into the multivariate model but did not reach statistical significance; blank multivariate cells indicate the variable was not entered into the multivariate model.

Table S3. Univariate and multivariate logistic regression analysis of factors associated with Low back WMSDs.

Variable	Category	n	Cases, n (%)	Univariate OR (95% CI)	P	Multivariate aOR (95% CI)	P
Hospital Grade	Secondary and primary hospitals (Ref.)	126	77 (61.1%)	1.00 (Reference)			
	Tertiary hospitals	274	187 (68.2%)	1.37 (0.88–2.12)	0.162		
Workload (patients/day)	<25 (Ref.)	127	82 (64.6%)	1.00 (Reference)			
	≥25	273	182 (66.7%)	1.10 (0.71–1.71)	0.680		
Participation in endoscopic procedures	Occasional (Ref.)	158	100 (63.3%)	1.00 (Reference)			
	Frequent	242	164 (67.8%)	1.22 (0.80–1.86)	0.356		
Age (years)	21–30 (Ref.)	115	72 (62.6%)	1.00 (Reference)			
	31–40	201	137 (68.2%)	1.28 (0.79–2.07)	0.316		
	41–50	68	48 (70.6%)	1.43 (0.75–2.73)	0.273		
	51–60	16	7 (43.8%)	0.46 (0.16–1.34)	0.155		
Gender	Male (Ref.)	48	27 (56.2%)	1.00 (Reference)			
	Female	352	237 (67.3%)	1.60 (0.87–2.96)	0.131		
BMI (kg/m²)	<24 (Ref.)	310	207 (66.8%)	1.00 (Reference)			
	24–27.9	72	47 (65.3%)	0.94 (0.55–1.60)	0.809		
	≥28	18	10 (55.6%)	0.62 (0.24–1.62)	0.332		
Nursing administration position	No (Ref.)	347	234 (67.4%)	1.00 (Reference)			
	Charge nurse	53	30 (56.6%)	0.63 (0.35–1.13)	0.123		
Nursing technical title	Nurse (Ref.)	44	28 (63.6%)	1.00 (Reference)			
	Nurse-in-charge	172	119 (69.2%)	1.28 (0.64–2.57)	0.482		

Variable	Category	n	Cases, n (%)	Univariate OR (95% CI)	P	Multivariate aOR (95% CI)	P
	Nurse practitioner	149	96 (64.4%)	1.04 (0.51–2.08)	0.923		
	Associate Chief Nurse or above	35	21 (60.0%)	0.86 (0.34–2.14)	0.741		
Work experience (years)	<5 (Ref.)	222	143 (64.4%)	1.00 (Reference)			
	6–10	111	79 (71.2%)	1.36 (0.83–2.24)	0.218		
	>10	67	42 (62.7%)	0.93 (0.53–1.63)	0.796		

Note: OR, odds ratio; aOR, adjusted odds ratio; CI, confidence interval; WMSDs, work-related musculoskeletal disorders. Only variables reaching P < 0.05 in the univariate analysis were entered into the multivariate model; "–" indicates the variable/category was entered into the multivariate model but did not reach statistical significance; blank multivariate cells indicate the variable was not entered into the multivariate model.

Table S4. Univariate and multivariate logistic regression analysis of factors associated with Shoulder WMSDs.

Variable	Category	n	Cases, n (%)	Univariate OR (95% CI)	P	Multivariate aOR (95% CI)	P
Hospital Grade	Secondary and primary hospitals (Ref.)	126	65 (51.6%)	1.00 (Reference)			
	Tertiary hospitals	274	167 (60.9%)	1.46 (0.96–2.24)	0.079		
Workload (patients/day)	<25 (Ref.)	127	84 (66.1%)	1.00 (Reference)		1.00 (Reference)	
	≥25	273	148 (54.2%)	0.61 (0.39–0.94)	0.025	0.66 (0.41–1.04)	0.073
Participation in endoscopic procedures	Occasional (Ref.)	158	77 (48.7%)	1.00 (Reference)		1.00 (Reference)	
	Frequent	242	155 (64.0%)	1.87 (1.25–2.82)	0.003	1.74 (1.05–2.90)	0.033
Age (years)	21–30 (Ref.)	115	63 (54.8%)	1.00 (Reference)		1.00 (Reference)	
	31–40	201	111 (55.2%)	1.02 (0.64–1.61)	0.940	0.86 (0.52–1.42)	0.561
	41–50	68	50 (73.5%)	2.29 (1.19–4.40)	0.013	–	–
	51–60	16	8 (50.0%)	0.83 (0.29–2.35)	0.719	0.67 (0.20–2.30)	0.530
Gender	Male (Ref.)	48	25 (52.1%)	1.00 (Reference)			
	Female	352	207 (58.8%)	1.31 (0.72–2.40)	0.377		
BMI (kg/m²)	<24 (Ref.)	310	189 (61.0%)	1.00 (Reference)			
	24–27.9	72	35 (48.6%)	0.61 (0.36–1.01)	0.057		
	≥28	18	8 (44.4%)	0.51 (0.20–1.33)	0.171		
Nursing administration	No (Ref.)	347	200 (57.6%)	1.00 (Reference)			

Variable	Category	n	Cases, n (%)	Univariate OR (95% CI)	P	Multivariate aOR (95% CI)	P
position							
	Charge nurse	53	32 (60.4%)	1.12 (0.62–2.02)	0.707		
Nursing technical title	Nurse (Ref.)	44	22 (50.0%)	1.00 (Reference)			
	Nurse-in-charge	172	104 (60.5%)	1.53 (0.79–2.97)	0.211		
	Nurse practitioner	149	84 (56.4%)	1.29 (0.66–2.54)	0.456		
	Associate Chief Nurse or above	35	22 (62.9%)	1.69 (0.68–4.18)	0.255		
Work experience (years)	<5 (Ref.)	222	110 (49.5%)	1.00 (Reference)		1.00 (Reference)	
	6–10	111	79 (71.2%)	2.51 (1.54–4.09)	<0.001	2.51 (1.45–4.34)	<0.001
	>10	67	43 (64.2%)	1.82 (1.04–3.21)	0.037	1.50 (0.75–3.01)	0.255

Note: OR, odds ratio; aOR, adjusted odds ratio; CI, confidence interval; WMSDs, work-related musculoskeletal disorders. Only variables reaching P < 0.05 in the univariate analysis were entered into the multivariate model; "–" indicates the variable/category was entered into the multivariate model but did not reach statistical significance; blank multivariate cells indicate the variable was not entered into the multivariate model.

Table S5. Univariate and multivariate logistic regression analysis of factors associated with Elbow WMSDs.

Variable	Category	n	Cases, n (%)	Univariate OR (95% CI)	P	Multivariate aOR (95% CI)	P
Hospital Grade	Secondary and primary hospitals (Ref.)	126	32 (25.4%)	1.00 (Reference)			
	Tertiary hospitals	274	58 (21.2%)	0.79 (0.48–1.29)	0.347		
Workload (patients/day)	<25 (Ref.)	127	31 (24.4%)	1.00 (Reference)			
	≥25	273	59 (21.6%)	0.85 (0.52–1.40)	0.533		
Participation in endoscopic procedures	Occasional (Ref.)	158	39 (24.7%)	1.00 (Reference)			
	Frequent	242	51 (21.1%)	0.81 (0.51–1.31)	0.398		
Age (years)	21–30 (Ref.)	115	22 (19.1%)	1.00 (Reference)		1.00 (Reference)	
	31–40	201	41 (20.4%)	1.08 (0.61–1.93)	0.786	0.87 (0.47–1.61)	0.664
	41–50	68	23 (33.8%)	2.16 (1.09–4.28)	0.027	4.04 (1.31–12.51)	0.015
	51–60	16	4 (25.0%)	1.41 (0.41–4.79)	0.583	1.10 (0.32–3.80)	0.884
Gender	Male (Ref.)	48	5 (10.4%)	1.00 (Reference)		1.00 (Reference)	
	Female	352	85 (24.1%)	2.74 (1.05–7.13)	0.039	3.11 (1.01–9.58)	0.049

Variable	Category	n	Cases, n (%)	Univariate OR (95% CI)	P	Multivariate aOR (95% CI)	P
BMI (kg/m²)	<24 (Ref.)	310	76 (24.5%)	1.00 (Reference)			
	24–27.9	72	12 (16.7%)	0.62 (0.31–1.21)	0.157		
	≥28	18	2 (11.1%)	0.38 (0.09–1.71)	0.210		
Nursing administration position	No (Ref.)	347	80 (23.1%)	1.00 (Reference)			
	Charge nurse	53	10 (18.9%)	0.78 (0.37–1.61)	0.497		
Nursing technical title	Nurse (Ref.)	44	12 (27.3%)	1.00 (Reference)			
	Nurse-in-charge	172	40 (23.3%)	0.81 (0.38–1.71)	0.579		
	Nurse practitioner	149	30 (20.1%)	0.67 (0.31–1.46)	0.315		
	Associate Chief Nurse or above	35	8 (22.9%)	0.79 (0.28–2.22)	0.654		
Work experience (years)	<5 (Ref.)	222	43 (19.4%)	1.00 (Reference)			
	6–10	111	31 (27.9%)	1.61 (0.95–2.75)	0.078		
	>10	67	16 (23.9%)	1.31 (0.68–2.51)	0.423		

Note: OR, odds ratio; aOR, adjusted odds ratio; CI, confidence interval; WMSDs, work-related musculoskeletal disorders. Only variables reaching P < 0.05 in the univariate analysis were entered into the multivariate model; "–" indicates the variable/category was entered into the multivariate model but did not reach statistical significance; blank multivariate cells indicate the variable was not entered into the multivariate model.

Table S6. Univariate and multivariate logistic regression analysis of factors associated with Wrist WMSDs.

Variable	Category	n	Cases, n (%)	Univariate OR (95% CI)	P	Multivariate aOR (95% CI)	P
Hospital Grade	Secondary and primary hospitals (Ref.)	126	51 (40.5%)	1.00 (Reference)			
	Tertiary hospitals	274	110 (40.1%)	0.99 (0.64–1.52)	0.950		
Workload (patients/day)	<25 (Ref.)	127	50 (39.4%)	1.00 (Reference)			
	≥25	273	111 (40.7%)	1.06 (0.69–1.62)	0.807		
Participation in endoscopic procedures	Occasional (Ref.)	158	58 (36.7%)	1.00 (Reference)			
	Frequent	242	103 (42.6%)	1.28 (0.85–1.93)	0.244		
Age (years)	21–30 (Ref.)	115	40 (34.8%)	1.00 (Reference)		1.00 (Reference)	
	31–40	201	81 (40.3%)	1.27 (0.79–2.04)	0.332	1.27 (0.79–2.04)	0.332

Variable	Category	n	Cases, n (%)	Univariate OR (95% CI)	P	Multivariate aOR (95% CI)	P
	41–50	68	35 (51.5%)	1.99 (1.08–3.66)	0.027	1.99 (1.08–3.66)	0.027
	51–60	16	5 (31.2%)	0.85 (0.28–2.62)	0.781	0.85 (0.28–2.62)	0.781
Gender	Male (Ref.)	48	16 (33.3%)	1.00 (Reference)			
	Female	352	145 (41.2%)	1.40 (0.74–2.65)	0.299		
BMI (kg/m²)	<24 (Ref.)	310	126 (40.6%)	1.00 (Reference)			
	24–27.9	72	31 (43.1%)	1.10 (0.66–1.85)	0.708		
	≥28	18	4 (22.2%)	0.42 (0.13–1.30)	0.131		
Nursing administration position	No (Ref.)	347	142 (40.9%)	1.00 (Reference)			
	Charge nurse	53	19 (35.8%)	0.81 (0.44–1.47)	0.484		
Nursing technical title	Nurse (Ref.)	44	13 (29.5%)	1.00 (Reference)			
	Nurse-in-charge	172	77 (44.8%)	1.93 (0.95–3.95)	0.070		
	Nurse practitioner	149	58 (38.9%)	1.52 (0.73–3.14)	0.259		
	Associate Chief Nurse or above	35	13 (37.1%)	1.41 (0.55–3.62)	0.476		
Work experience (years)	<5 (Ref.)	222	81 (36.5%)	1.00 (Reference)			
	6–10	111	51 (45.9%)	1.48 (0.93–2.35)	0.097		
	>10	67	29 (43.3%)	1.33 (0.76–2.31)	0.316		

Note: OR, odds ratio; aOR, adjusted odds ratio; CI, confidence interval; WMSDs, work-related musculoskeletal disorders. Only variables reaching P < 0.05 in the univariate analysis were entered into the multivariate model; "–" indicates the variable/category was entered into the multivariate model but did not reach statistical significance; blank multivariate cells indicate the variable was not entered into the multivariate model.

Table S7. Univariate and multivariate logistic regression analysis of factors associated with Hip WMSDs.

Variable	Category	n	Cases, n (%)	Univariate OR (95% CI)	P	Multivariate aOR (95% CI)	P
Hospital Grade	Secondary and primary hospitals (Ref.)	126	24 (19.0%)	1.00 (Reference)			
	Tertiary hospitals	274	69 (25.2%)	1.43 (0.85–2.41)	0.179		
Workload (patients/day)	<25 (Ref.)	127	37 (29.1%)	1.00 (Reference)			
	≥25	273	56 (20.5%)	0.63 (0.39–1.02)	0.059		
Participation in	Occasional (Ref.)	158	31 (19.6%)	1.00 (Reference)			

Variable	Category	n	Cases, n (%)	Univariate OR (95% CI)	P	Multivariate aOR (95% CI)	P
endoscopic procedures							
	Frequent	242	62 (25.6%)	1.41 (0.87–2.30)	0.166		
Age (years)	21–30 (Ref.)	115	25 (21.7%)	1.00 (Reference)			
	31–40	201	47 (23.4%)	1.10 (0.63–1.91)	0.738		
	41–50	68	18 (26.5%)	1.30 (0.65–2.60)	0.466		
	51–60	16	3 (18.8%)	0.83 (0.22–3.15)	0.785		
Gender	Male (Ref.)	48	7 (14.6%)	1.00 (Reference)			
	Female	352	86 (24.4%)	1.89 (0.82–4.38)	0.135		
BMI (kg/m²)	<24 (Ref.)	310	74 (23.9%)	1.00 (Reference)			
	24–27.9	72	15 (20.8%)	0.84 (0.45–1.57)	0.583		
	≥28	18	4 (22.2%)	0.91 (0.29–2.85)	0.873		
Nursing administration position	No (Ref.)	347	85 (24.5%)	1.00 (Reference)			
	Charge nurse	53	8 (15.1%)	0.55 (0.25–1.21)	0.136		
Nursing technical title	Nurse (Ref.)	44	11 (25.0%)	1.00 (Reference)			
	Nurse-in-charge	172	42 (24.4%)	0.97 (0.45–2.08)	0.936		
	Nurse practitioner	149	35 (23.5%)	0.92 (0.42–2.01)	0.836		
	Associate Chief Nurse or above	35	5 (14.3%)	0.50 (0.16–1.61)	0.244		
Work experience (years)	<5 (Ref.)	222	46 (20.7%)	1.00 (Reference)			
	6–10	111	30 (27.0%)	1.42 (0.83–2.41)	0.197		
	>10	67	17 (25.4%)	1.30 (0.69–2.46)	0.420		

Note: OR, odds ratio; aOR, adjusted odds ratio; CI, confidence interval; WMSDs, work-related musculoskeletal disorders. Only variables reaching P < 0.05 in the univariate analysis were entered into the multivariate model; "–" indicates the variable/category was entered into the multivariate model but did not reach statistical significance; blank multivariate cells indicate the variable was not entered into the multivariate model.

Table S8. Univariate and multivariate logistic regression analysis of factors associated with Knee WMSDs.

Variable	Category	n	Cases, n (%)	Univariate OR (95% CI)	P	Multivariate aOR (95% CI)	P
Hospital Grade	Secondary and primary hospitals (Ref.)	126	47 (37.3%)	1.00 (Reference)			

Variable	Category	n	Cases, n (%)	Univariate OR (95% CI)	P	Multivariate aOR (95% CI)	P
	Tertiary hospitals	274	115 (42.0%)	1.22 (0.79–1.88)	0.377		
Workload (patients/day)	<25 (Ref.)	127	57 (44.9%)	1.00 (Reference)			
	≥25	273	105 (38.5%)	0.77 (0.50–1.18)	0.224		
Participation in endoscopic procedures	Occasional (Ref.)	158	52 (32.9%)	1.00 (Reference)		1.00 (Reference)	
	Frequent	242	110 (45.5%)	1.70 (1.12–2.58)	0.013	1.69 (1.01–2.80)	0.044
Age (years)	21–30 (Ref.)	115	35 (30.4%)	1.00 (Reference)		1.00 (Reference)	
	31–40	201	82 (40.8%)	1.58 (0.97–2.56)	0.067	1.26 (0.73–2.17)	0.407
	41–50	68	39 (57.4%)	3.07 (1.65–5.73)	<0.001	4.75 (1.74–12.97)	0.002
	51–60	16	6 (37.5%)	1.37 (0.46–4.07)	0.569	1.15 (0.33–4.02)	0.824
Gender	Male (Ref.)	48	13 (27.1%)	1.00 (Reference)		1.00 (Reference)	
	Female	352	149 (42.3%)	1.98 (1.01–3.86)	0.047	1.73 (0.82–3.66)	0.153
BMI (kg/m²)	<24 (Ref.)	310	128 (41.3%)	1.00 (Reference)			
	24–27.9	72	28 (38.9%)	0.90 (0.54–1.53)	0.709		
	≥28	18	6 (33.3%)	0.71 (0.26–1.94)	0.506		
Nursing administration position	No (Ref.)	347	142 (40.9%)	1.00 (Reference)			
	Charge nurse	53	20 (37.7%)	0.87 (0.48–1.59)	0.660		
Nursing technical title	Nurse (Ref.)	44	16 (36.4%)	1.00 (Reference)			
	Nurse-in-charge	172	78 (45.3%)	1.45 (0.73–2.88)	0.285		
	Nurse practitioner	149	54 (36.2%)	0.99 (0.49–2.00)	0.988		
	Associate Chief Nurse or above	35	14 (40.0%)	1.17 (0.47–2.91)	0.741		
Work experience (years)	<5 (Ref.)	222	73 (32.9%)	1.00 (Reference)		1.00 (Reference)	
	6–10	111	56 (50.5%)	2.08 (1.30–3.31)	0.002	1.85 (1.09–3.12)	0.022
	>10	67	33 (49.3%)	1.98 (1.14–3.45)	0.016	1.28 (0.65–2.52)	0.471

Note: OR, odds ratio; aOR, adjusted odds ratio; CI, confidence interval; WMSDs, work-related musculoskeletal disorders. Only variables reaching P < 0.05 in the univariate analysis were entered into the multivariate model; "–" indicates the variable/category was entered into the multivariate model but did not reach statistical significance; blank multivariate cells indicate the variable was not entered into the multivariate model.

Table S9. Univariate and multivariate logistic regression analysis of factors associated with Ankle WMSDs.

Variable	Category	n	Cases, n (%)	Univariate OR (95% CI)	P	Multivariate aOR (95% CI)	P
Hospital Grade	Secondary and primary hospitals (Ref.)	126	40 (31.7%)	1.00 (Reference)			
	Tertiary hospitals	274	110 (40.1%)	1.44 (0.92–2.25)	0.108		
Workload (patients/day)	<25 (Ref.)	127	55 (43.3%)	1.00 (Reference)			
	≥25	273	95 (34.8%)	0.70 (0.45–1.07)	0.102		
Participation in endoscopic procedures	Occasional (Ref.)	158	49 (31.0%)	1.00 (Reference)		1.00 (Reference)	
	Frequent	242	101 (41.7%)	1.59 (1.04–2.43)	0.031	1.59 (1.04–2.43)	0.031
Age (years)	21–30 (Ref.)	115	47 (40.9%)	1.00 (Reference)			
	31–40	201	73 (36.3%)	0.83 (0.52–1.32)	0.423		
	41–50	68	25 (36.8%)	0.84 (0.45–1.56)	0.583		
	51–60	16	5 (31.2%)	0.66 (0.21–2.02)	0.464		
Gender	Male (Ref.)	48	13 (27.1%)	1.00 (Reference)			
	Female	352	137 (38.9%)	1.72 (0.88–3.36)	0.115		
BMI (kg/m²)	<24 (Ref.)	310	118 (38.1%)	1.00 (Reference)			
	24–27.9	72	24 (33.3%)	0.81 (0.47–1.40)	0.455		
	≥28	18	8 (44.4%)	1.30 (0.50–3.39)	0.589		
Nursing administration position	No (Ref.)	347	135 (38.9%)	1.00 (Reference)			
	Charge nurse	53	15 (28.3%)	0.62 (0.33–1.17)	0.140		
Nursing technical title	Nurse (Ref.)	44	17 (38.6%)	1.00 (Reference)			
	Nurse-in-charge	172	70 (40.7%)	1.09 (0.55–2.15)	0.804		
	Nurse practitioner	149	56 (37.6%)	0.96 (0.48–1.91)	0.899		
	Associate Chief Nurse or above	35	7 (20.0%)	0.40 (0.14–1.11)	0.078		
Work experience (years)	<5 (Ref.)	222	77 (34.7%)	1.00 (Reference)			
	6–10	111	46 (41.4%)	1.33 (0.83–2.13)	0.229		
	>10	67	27 (40.3%)	1.27 (0.73–2.23)	0.402		

Note: OR, odds ratio; aOR, adjusted odds ratio; CI, confidence interval; WMSDs, work-related musculoskeletal disorders. Only variables reaching P < 0.05 in the univariate analysis were entered into the multivariate model; "–" indicates the variable/category was entered into the multivariate model but did not reach statistical significance; blank multivariate cells indicate the variable was not entered into the multivariate model.
